# Increased Substance Use among Nurses during the COVID-19 Pandemic

**DOI:** 10.3390/ijerph20032674

**Published:** 2023-02-02

**Authors:** Eamonn Arble, Dana Manning, Bengt B. Arnetz, Judith E. Arnetz

**Affiliations:** 1 Department of Psychology, Eastern Michigan University, Ypsilanti, MI 48197, USA; 2 Department of Family Medicine, College of Human Medicine, Michigan State University, Grand Rapids, MI 49503, USA

**Keywords:** nurses, cognitive failure, COVID-19, trauma

## Abstract

There is growing evidence that the COVID-19 pandemic has had a severe impact on the nursing profession worldwide. Occupational strain has disrupted nurses’ emotional wellbeing and may have led to negative coping behaviors, such as increased substance use, which could impair cognitive functioning. The aim of this study was to examine whether increased substance use in a sample of U.S. nurses during the pandemic was related to greater workplace cognitive failure. An online questionnaire was administered in May 2020 to Michigan nurses statewide via three nursing organizations (n = 695 respondents). A path model was used to test the direct effects of reported increased substance use on workplace cognitive failure and via parallel psychological mediators. The model had excellent fit to the observed data, with statistically significant, unique mediating effects of greater symptoms of anxiety (b = 0.236, z = 2.22, *p* = 0.027), posttraumatic stress disorder (b = 0.507, z = 4.62, *p* < 0.001) and secondary trauma (b = 1.10, z = 2.82, *p* = 0.005). Importantly, the direct effect of increased substance use on workplace cognitive failure was not statistically significant independent of the mediators (b = 0.133, z = 0.56, *p* = 0.576; 95% confidence interval: −0.33, 0.60). These results point to the importance of further delineating the mechanistic pathways linking adverse stress to workplace cognitive failure. As we emerge from the pandemic, healthcare systems should focus resources on supporting cognitive health by addressing the psychological and emotional welfare of nurses, many of whom may be struggling with residual trauma and increased substance use.

## 1. Introduction

The COVID-19 global pandemic has fundamentally changed the experience of nurses throughout the world. There has been growing evidence of the severe negative impact the pandemic has had on nurses’ health and wellbeing [1]. Nurses have been forced to confront contagion, understaffing, isolation, lack of governmental and public support, and countless other practical, emotional, and physical challenges that have deeply affected them [2]. A recent systematic review including over 30,000 participants found that nurses are the most affected healthcare providers within the pandemic [3]. The consequences of this ongoing strain include depression, anxiety, insomnia [4], psychological distress [5], post-traumatic stress [6], and burnout [7]. Recent empirical investigations have also begun to identify the consequences of the pandemic on nurses’ workplace cognitive functioning, and in particular, instances when nurses experience cognitive failure [8]. This is a key variable for researchers to examine, as cognitive failure is an essential predictor of nursing performance, patient outcomes, and the overall wellbeing of nurses [9].

### 1.1. Workplace Cognitive Failure

Nursing is a highly sophisticated occupation, requiring a knowledge of medical procedures, an ability to navigate interpersonal styles (of both patients, physicians, and other colleagues), and the management of multiple time-sensitive and critical demands. At any given moment, a nurse may be charged with carrying out a medical procedure with an uncooperative patient while simultaneously considering an upcoming activity that must be completed within the hour. Under these constraints, errors are to some degree inevitable. Broadbent and colleagues introduced the concept of “cognitive failure” to describe such errors in perception, memory, or action [10]. The nurse who completes their current task while forgetting the upcoming task to be completed within the hour is said to have experienced cognitive failure. Of course, in the healthcare profession, cognitive failures can have severe consequences.

In the years after Broadbent’s original work, research on the concept of cognitive failure grew to include dispositional predictors of experiencing cognitive failure [11], its application to non-healthcare related professions [12], and specific measures designed to assess the experience of cognitive failure at work [13]. Critically, this research began to identify the contextual and occupational factors that predicted the experience of cognitive failure. Among nurses specifically, increased levels of stress and the need to suddenly switch between tasks (i.e., workflow interruption) are associated with the experience of increased rates of cognitive failure [14,15]. These findings appear sensible. As tasks grow in complexity, and as the individuals completing those tasks become more affectively strained and cognitively burdened, an increase in errors is a natural possibility.

The potential link between workplace cognitive failure and nursing performance took on particular significance during the course of the COVID-19 pandemic. As previously noted, during the pandemic, nurses experienced dramatically increased levels of occupational stress [16] and increasingly demanding and complex occupational strains [17]. They faced increased patient morbidity [18] and endured emotional fatigue and elevated trauma exposure [19]. These elements point to the very conditions under which rates of cognitive failure could increase, which in turn may lead to an increase in adverse patient outcomes [15]. The result may be a vicious cycle in which nurses are emotionally exhausted and overworked and thus prone to cognitive failure, which may result in poor patient outcomes, which in turn leaves nurses feeling even more emotionally strained and vulnerable. As the strains and traumatic exposures for nurses intensify, there is concern that substance use may be a growing pathology within the field [20].

### 1.2. Substance Use

The link between the experience of stressful or traumatic events and the use of substances has been well established [21]. In its most severe forms, comorbidity between the diagnoses of posttraumatic stress disorder (PTSD) and substance use disorder (SUD) is significant, with alcohol use disorder (AUD) being the most commonly occurring SUD comorbidity among samples of individuals diagnosed with PTSD [22,23]. Many explanations for this relationship have been proposed. Some models suggest that the use of substances increases the risk of experiencing traumatic or stressful events by placing individuals in more precarious situations and contexts [24]. A nurse whose substance use caused them to commit a severe error at work would fit such a description—they are now forced to confront the emotional and practical consequences of their mistake. Other models suggest that individuals experiencing the symptoms of trauma may seek relief through self-medication, using substances such as alcohol to blunt the experience of anxiety that they may be enduring [25]. A nurse who struggles to sleep after their shift and turns to alcohol to relax would fit such a description.

A full exploration of the overlap between trauma and substance use is beyond the scope of this paper, as is a complete discussion of the variety of conceptual models explaining it. However, it is noteworthy that a wide body of empirical and theoretical research on the topic has identified the central roles of emotion regulation and coping styles. Individuals who struggle to regulate their emotions are more likely to report symptoms of posttraumatic stress and substance use [26,27]. Furthermore, individuals with maladaptive coping styles, and in particular, the use of substances as a form of avoidant coping, generally demonstrate worse outcomes regarding health, relationships, and occupational performance [28].

Accurate and comprehensive data on the rates of substance use among nurses are hard to obtain. Early research on the subject suggested that nurses were less likely to use some substances (e.g., cocaine) than the general population, but were significantly more likely than the general population to engage in prescription drug misuse [29]. More recent research indicates that substance use rates among nurses are largely comparable to the general population [30], though some have raised concerns that these results may reflect a degree of underreporting [31]. Nonetheless, taking these conservative estimates, as many as one in five nurses may engage in maladaptive substance use [32]. Alarmingly, the rates of substance use may have increased as a result of the pandemic [33,34].

Both the experience of trauma and the use of substances have been linked to increased rates of cognitive failure. Individuals in high-stress situations are more likely to experience cognitive failure, evidenced by an increased error rate in cognitive tasks [35]. Individuals diagnosed with a psychological disorder are more susceptible to cognitive failures, particularly those with posttraumatic stress disorder [36]. This may be attributed to disruptions in executive functioning and the cognitive burden of experiencing intrusive memories [36]. The disruption of sleep as a result of trauma and substance use may also result in cognitive failure; sleep quality has an inverse relationship with both reported stress levels and cognitive failure occurrences [8]. Those who exhibit symptoms of insomnia are more likely to experience cognitive failure, a relationship that may be mediated by individual stress [37]. In general, as reported symptoms of psychopathology increase, so does the frequency of cognitive failure [35]. 

### 1.3. Present Study

In 2021, Arnetz and colleagues conducted a large study of almost 700 nurses to identify factors associated with workplace cognitive failure during the early phases of the COVID-19 pandemic [8]. There were several important findings of that research, two of which the present study will directly build upon. First, cognitive failure was a commonly endorsed problem among nurses surveyed, with over 75% of nurses reporting some degree of cognitive failure. Second, although there were direct effects between cognitive failure and exposure to COVID-19 patients and the availability of personal protective equipment (PPE), respectively, the effects were mediated by a range of psychosocial factors. These results speak to not only the prevalence of cognitive failure, but further, the clear importance of coping strategies in understanding the emergence of cognitive failure among nurses.

The present study expands upon the results from Arnetz et al. [8] by examining a specific subsample within the data. Namely, nurses who responded to survey items regarding substance use were selected for an analysis of the effect of reported increases in substance use on workplace cognitive failure. This study aims to examine whether increased substance use was related to workplace cognitive failure, and whether psychological and emotional symptoms mediated that relationship. Nurses who reported an increase in alcohol, marijuana or other drug use during the COVID-19 pandemic were compared to nurses who reported no change, decrease, or no current substance use to test the following hypotheses:

**H1.**  *Reported increased substance use during the COVID-19 pandemic will be associated with greater workplace cognitive failure*.

**H2.**  *The effects of increased substance use on workplace cognitive failure will be partially mediated by intervening psychological and emotional symptoms associated with the experience of trauma*.

**H3.**  *Based on the unprecedented event of the COVID-19 pandemic, the proportion of mediated effects will be largely accounted for by trauma symptoms specifically (PTSD and secondary trauma) as compared to other hypothesized mediators of depression, anxiety, and disrupted sleep*.

## 2. Materials and Methods

### 2.1. Design

During the height of the pandemic (May of 2020), an online survey was distributed to a sample of nurses in the state of Michigan. The survey was hosted on the Qualtrics platform. The study was reviewed by the Institutional Review Board of Michigan State University and was determined exempt (MSU Study ID: STUDY00004459).

### 2.2. Participants

Participants in the study were drawn from professional nursing organizations within the state of Michigan: the American Nurses Association (ANA) Michigan; the Michigan Organization of Nurse Leaders (MONL); the Coalition of Michigan Organizations of Nursing (COMON). ANA Michigan electronically delivered a link to the survey directly to its members. A snowball recruitment method was utilized among MONL and COMON, where members were asked to distribute the survey link to members within their organization. This sample was characterized in a prior publication [8], and a subsample that responded to survey items regarding substance use is included in the current report.

### 2.3. Measures

#### 2.3.1. Patient Health Questionnaire–9 (PHQ-9)

Symptoms of depression were measured with the PHQ-9 [38], a nine-item self-report measure. Respondents are asked to rate the frequency of their experience with a variety of depressive symptoms. Scores range from 0 to 27, with higher scores indicating greater depressive symptoms. Cronbach’s alpha for the PHQ-9 was 0.88 in the current study.

#### 2.3.2. Generalized Anxiety Disorder–7 (GAD-7)

Symptoms of anxiety were measured with the GAD-7 [39], a seven-item self-report measure. Respondents are asked to rate the frequency of their experience with a variety of anxiety symptoms. Scores range from 0 to 21, with higher scores indicating greater anxiety symptoms. Cronbach’s alpha for the GAD-7 in the current study was 0.93.

#### 2.3.3. PTSD Checklist (PCL-6)

PTSD symptoms were measured with the PCL-6, a six-item self-report measure; the PCL-6 is an abbreviated version of the full, 20-item PTSD Checklist [40]. Respondents are asked to report the intensity of their experience with a range of PTSD symptoms. Scores range from 6 to 30, with higher scores indicating greater PTSD symptoms. Cronbach’s alpha for the PCL-6 was 0.88 in the current study.

#### 2.3.4. Stress and Sleep Quality

The experience of stress and the quality of sleep were assessed through validated single-item 0–10 visual analogue scales (VAS). Responders were asked: “How do you rate your current stress level?” and “How do you rate your current sleep quality?" The effective use of single-item VAS for these constructs has been demonstrated in previous research [8,41].

#### 2.3.5. Secondary Trauma

Secondary trauma was measured via a single item from the Compassion Fatigue subscale of the ProQuol [42]. Respondents were asked to use a five-point scale to respond to the following: “I feel as though I am experiencing the trauma of the patients I care for”. Higher scores indicate greater levels of secondary trauma.

#### 2.3.6. Work-Related Exhaustion

Work-related exhaustion was measured by a three-item subscale from the Quality Work Competence (QWC) questionnaire [43], a standardized self-report measure. Respondents were asked to rate the frequency with which they experienced work-related exhaustion (e.g., “I feel tired when I think about work”). Scores range from 3 to 15, with higher scores indicating greater work-related exhaustion. Cronbach’s alpha for the QWC scale items was 0.92 in the current study.

#### 2.3.7. Workplace Cognitive Failure Scale (WCFS)

Cognitive failure was measured by the WCFS [13], a 15-item self-report measure. Respondents rate the frequency with which they experience instances of cognitive failure. Only the composite score was used in the present study; this score ranges from 15 to 75, with higher scores indicating greater frequency of cognitive failure. Cronbach’s alpha for the WCFS was 0.91 in the current study.

### 2.4. Statistical Analysis

Prior to hypothesis testing, descriptive analyses of the sample responding to relevant variables of substance use were completed with SPSS (version 28, IBM, Armonk, NY, USA) [44] to examine frequencies of participant endorsement of substance use change and Spearman rho correlations of all variables. Responses to questionnaire items regarding substance use of alcohol, marijuana, and illicit drugs were recoded to compare nurses who reported more substance use during the COVID-19 pandemic as compared to all other responses (i.e., no change, decreased use, and no reported use). All other constructs were represented by single summary scale scores.

Hypotheses were tested with a path model estimated in MPlus (Version 7, Muthén, L.K. and Muthén, B.O., Los Angeles, CA, USA) [45] constructed to test the effect of increased substance use on workplace cognitive failure via multiple, correlated mediators: depression, anxiety, PTSD, stress, sleep quality, secondary trauma and work-related exhaustion. Participant age was coded as a categorical variable for 45 years and older or younger, which was included as a covariate. Because several of the hypothesized mediating variables were measured on ordinal scales, the model was estimated with robust maximum likelihood. Model fit was evaluated by a compendium of accepted indices [46,47]: chi-square non-significance, comparative fit index (CFI) > 0.9; root mean square error of approximation (RMSEA) < 0.08; and standardized root mean squared residual (SRMR) < 0.05 together indicated excellent model fit. Hypothesized effects of reported increased substance use were tested in a model that included a direct effect on workplace cognitive failure and tests of mediation with indirect effects and bootstrapped 95% confidence intervals [48]. Indirect effects were evaluated for statistical significance with a Sobel z-test, and effect sizes were further described with 95% confidence intervals (CI) and proportion of total effect of increased substance use.

## 3. Results

### 3.1. Description of the Sample

A total of 695 nurses initiated the survey, of which 615 completed descriptive demographic and substance use questions (Table 1). Among those responding to substance use items and reporting an increase in substance use (*n* = 198; 32%), *n* = 159 (25.9%) reported a current increase in drinking, *n* = 16 (2.6%) an increase in marijuana use, and *n* = 23 (3.7%) an increase in other drug use as compared to before the COIVD-19 pandemic. In further analysis, individuals who reported an increase in any of these substances were compared to the remainder of the sample (i.e., no use, decreased use, and no change in use).

Model estimation for hypothesis testing required complete data on the predictor variables, and of the available sample, n = 613 had complete data on these variables; data were found to be missing at random (Little’s χ^2^ (3) = 35.49, *p* = 0.31) and, therefore, listwise deletion of cases missing these variables was expected to introduce negligible bias into the model-based estimates [49]. A summary of scale score distributions and bivariate correlations among variables of interest included in the analysis are reported in Table 2.

### 3.2. Psycho-Affective Symptoms Mediate Effects of Increased Substance Use on Workplace Cognitive Failure

**Figure 1:**
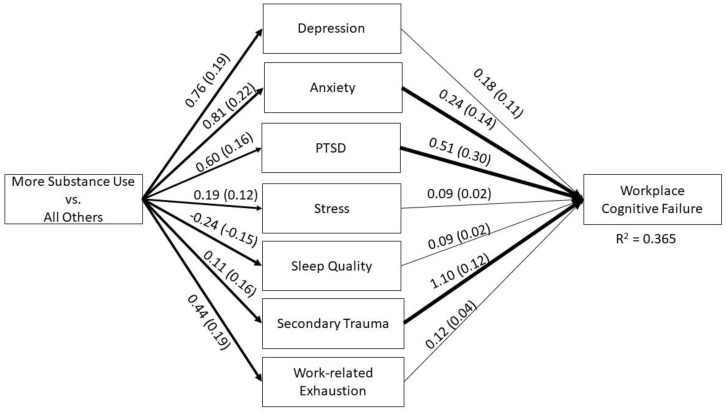
A summary path diagram of the hypothesized model testing the effects of reported increased substance use on workplace cognitive failure via multiple psychological mediators. Unstandardized coefficients (standardized coefficients) are reported for each path; bolded paths indicate statistical significance (*p* < 0.05). The cumulative model accounted for 37% of the variability in the outcome.

The hypothesized model testing the effects of reported increased substance use on workplace cognitive failure via parallel psychological mediators had excellent fit to the observed data: χ^2^ (1) = 2.89, *p* = 0.089, CFI = 0.999, RMSEA = 0.056, and SRMR = 0.015. Categorical age was included as a covariate to the distal dependent variable and all mediators, except for depression, which was found to be a negligible effect and was constrained to zero (see Figure 1 for a path model diagram). The cumulative model accounted for 37% of the variance in workplace cognitive failure (R^2^ = 0.365, z = 11.27, *p* < 0.001). The total effect of reported increase in substance use on WCFS was significant (unstandardized effect = 0.94, z = 3.46, *p* = 0.001), indicating those who reported increased use during the COVID-19 pandemic experienced greater cognitive failure as compared to those who did not. The effect of increased substance use was predominantly explained by the intervening psychological variables, and the unique effect of substance use alone was not statistically significant independent of the mediators (b = 0.133, z = 0.56, *p* = 0.576; 95% CI: −0.33, 0.60).

Of the cumulative effect of increased substance use on workplace cognitive failure, 86.48% was mediated. Although all hypothesized factors contributed to the model, not all paths were statistically significant: greater anxiety (b = 0.236, z = 2.22, *p* = 0.027), greater PTSD symptoms (b = 0.507, z = 4.62, *p* < 0.001) and greater experience of secondary trauma (b = 1.10, z = 2.82, *p* = 0.005) were the only mediators that had statistically significant, unique effects. These three variables were also the only variables found to statistically significantly mediate the effects of increased reported substance use (Table 3). In rank order, PTSD accounted for the largest portion of the cumulative effect (31.10%, *p* = 0.005), followed by anxiety (19.41%, *p* < 0.05) and secondary trauma (12.50%, *p* = 0.023).

Although depression symptoms contributed to the model and accounted for approximately 14% of the effect of increased reported substance use, this indirect effect was not supported by the significance test or the 95% confidence intervals that overlapped with zero. On the whole, reported increased substance use during the COVID-19 pandemic was associated with greater workplace cognitive failure, and this was in the majority explained by increased experiences of trauma and anxiety symptoms.

## 4. Discussion

The experience of nurses during the pandemic is an essential topic for investigation. As an occupational group, they endured a dramatic increase in stressful experiences and a simultaneous rise in professional demands, leaving them uniquely vulnerable to the emergence of maladaptive coping strategies (i.e., substance use) and the occurrence of workplace cognitive failure. The present study sought to add to the extant literature by examining a subsample of nurses who reported increases in their substance use during the pandemic, testing the effects of substance use on workplace cognitive failure directly as well as via parallel psychological mediators. Nurses who reported increased substance use during the COVID-19 pandemic experienced greater cognitive failure compared to those who did not. However, the direct effect of increased substance use on cognitive failure was not significant and was instead predominantly explained by the intervening psychological variables. In particular, PTSD symptoms, anxiety, and secondary trauma emerged as the only mediators with statistically significant, unique effects.

This current sample was described in a prior publication [8]; among the original sample, approximately one-third of the surveyed nurses reported an increase in substance use during the pandemic. It is among this subsample that the present research is focused. Although there was undoubtedly variability in the extent to which nurses increased their substance use (some increases may have been quite mild, while others may have been extreme), these rates of increased substance use nonetheless point to a troubling finding. Increased substance use among nurses has the potential to result in harm to the nurses themselves, their patients, as well as their colleagues. Furthermore, although the anonymous nature of the survey may have offered sufficient confidentiality for the responding nurses to answer honestly, the legal and employment implications of disclosing substance use may have nonetheless resulted in underestimation of current usage [50]. Regardless, these findings indicate that substance use among nurses, particularly in times of great distress, should be carefully monitored.

Given that stressful occupational circumstances are predictive of substance use among nurses [51], and that stress and substance use have been found to be associated with increased cognitive failures [36], the strong relationship between substance use and workplace cognitive failure in the present sample appears reasonable. Although some nurses may have increased their substance use during the pandemic in a responsible manner, the combination of increased stress and substance use is a potentially dangerous one. Symptoms of intoxication, withdrawal, and faulty judgment may be a direct result of recent or contemporaneous substance use, a worrisome prospect that healthcare systems have struggled with even prior to the onset of the pandemic and its accompanying challenges [52]. Substances are certainly capable of producing the psychophysiological preconditions to dramatically increase the frequency and potency of workplace cognitive failures within a medical context, and given the frequency with which nurses reported an increase in substance use, the possibility of climbing rates of workplace cognitive failures may present an ongoing challenge.

However, a full understanding of the relationship between substance use and cognitive failure requires a deeper exploration of the emotional distress experienced by nurses, which some researchers have referred to as an epidemic of its own [32]. Emotional distress has been a noted challenge among nurses, with research indicating relatively high rates of depression and anxiety [53]. As noted previously, there is a robust literature identifying the connection between substance use and psychopathology. For instance, a nurse feeling on edge could engage in prescription drug abuse in order to relax; for such an individual, the misused prescription could be the source of their cognitive errors, but it is also plausible that the underlying clinical distress prompting their prescription misuse significantly contributes to their experience of cognitive failure. In other words, pronounced symptoms of anxiety could drive both prescription drug misuse and workplace cognitive failure. Indeed, the presented data suggest just such a model, and further longitudinal study is needed to determine the leading cause and potential bidirectional effect.

While all of the clinical issues measured were significantly correlated with workplace cognitive failure and contributed to the model, PTSD, anxiety, and secondary trauma proved to be the statistically significant, unique mediators. Importantly, the experiences of anxiety, trauma, and secondary trauma share a good deal of conceptual and clinical overlap, with some research suggesting the existence of a shared vulnerability model across the experiences [54]. PTSD is defined as the development of clinical symptoms subsequent to exposure to a traumatic event. The symptoms of PTSD are organized into several clusters [55], including intrusive symptoms associated with the event (e.g., intrusive memories of the event), avoidance of stimuli associated with the event (e.g., avoiding people associated with the event), negative alteration in thoughts and mood (e.g., persistent feelings of shame), and changes in stress arousal and reactivity (e.g., heightened startle response). Generalized anxiety disorder (GAD) is characterized by a pervasive state of anxiety and worry concerning a number of events or activities [55]. Unlike PTSD, GAD symptoms are not associated with a specific event; however, the two pathologies share the symptoms of avoidance, irritability, and anxiety.

Although not a diagnosis, secondary trauma is a construct highly related to PTSD. In secondary trauma, individuals are indirectly exposed to traumatic events, often taking on the pain and trauma of the people in their care. This phenomenon has been widely recognized among nurses, with some estimates identifying prevalence rates as high as 60% [56]. The experience of secondary trauma will of course reflect the nature of the work engaged in, leaving some nursing specialties even more vulnerable to the experience if their specialty requires more frequent or enduring exposure to patients suffering from trauma [57]. Unfortunately, trauma exposure, and, therefore, the risk of secondary trauma, was a highly common experience for nurses working during the pandemic [2], extending across healthcare settings [58]. Not only were nurses forced to confront the terror of patients succumbing to the new virus, but they were further left to contend with patients having to suffer in isolation, with nurses being among the very few individuals allowed close contact. The emotional burden was undoubtedly tremendous. Secondary trauma is also a component of compassion fatigue, which has in prior research been associated with an increase in substance use among nurses [59].

That these clusters of symptoms—PTSD, anxiety, and secondary trauma—operated as mediators in the relationship between increased substance use and workplace cognitive failure raises the alarming possibility that the experience of the pandemic was, in a real sense, a trauma for the nursing profession. Nurses were faced with increasing practical demands and emotional hardships; patient deaths, fears of illness exposure, increased workload, and operating in an uncertain world all may have contributed to the emergence of symptoms of trauma and anxiety. This would suggest that increased substance use among nurses is not merely a reflection of increased stress, but instead may further reflect the development of more serious psychological concerns. While clinical psychological symptoms are challenging on their own, when combined with substance use, the effects can be deadly for practicing nurses [32]. As the pandemic subsides, the welfare of nurses appears to be a primary concern for medical systems, and evidence of notable changes in functioning might belie a more serious issue to be addressed.

### Limitations

The results of this study should be considered for its strengths and limitations. The cross-sectional design of the study does not allow testing the direction of causality within the model; however, a strength of the approach is considering the proportional contribution of parallel psychological mediators. The study utilized a convenience sample that, while large, may not be representative of the nursing profession at a national level. Relatedly, due to the use of snowball sampling, an exact response rate could not be calculated. Finally, the measures included were all self-report and, where possible, emphasized efficiency. A more comprehensive and multi-method assessment could likely provide additional nuance to the present findings. For example, measures of clinical functioning that included biological data, controls for defensive responding, and additional non-self-report procedures (e.g., performance-based assessment data) could likely provide a fuller understanding of the underlying clinical dynamics.

## 5. Conclusions

The present results suggest that in the coming years, the emotional wellbeing of nurses should be a topic of focus for both researchers and administrators. In addition to a focus on substance use and cognitive failure, symptoms of anxiety and trauma may be of particular importance. Some nurses may have experienced the “Covid years” as an extended period of intense emotional pain, complicated by direct or indirect traumatic events. Many nurses may be suffering with residual symptoms of either trauma or anxiety, which, as the present data indicate, could function as part of an ongoing cycle of substance use and cognitive failure, negatively impacting not only nurses’ health, but also their ability to provide quality care for their patients. Healthcare systems should thus dedicate resources to supporting the psychological and emotional welfare of nurses. In part, this may require psychoeducation: some amount of distress and negative affect is to be expected within the nursing profession, but this is to be distinguished from psychopathology, trauma, and potentially dangerous substance use. To the extent possible, hospitals may wish to promote a culture of self-care and support, bolstered by access to tools and applications that nurses may utilize to assess their current levels of emotional distress and their need for additional assistance. Furthermore, if substance use operates as a coping mechanism, administrators would do well to provide interventions and recommendations that offer more adaptive coping strategies. This may take the form of referrals to available internal and community resources, or system-specific interventions that have demonstrated promising empirical results [60]. Similarly, additional information about positive coping behaviors and psycho-social support that has been found to bolster resiliency to trauma [61] would be useful targets for future studies to determine avenues to assist nurses while deterring substance use as a coping behavior.

## Figures and Tables

**Table 1:** Demographic description of the sample.

Variable	*n*	%
Self-identified female	574	93.3
Race/Ethnicity		
Non-Hispanic White	541	88
Black and African American	34	5.5
Asian	15	2.4
Age		
18–34 years	123	20
35–44 years	151	24.6
45–54 years	139	22.6
55–64 years	157	25.5
65 years and older	43	7
Current Work Schedule		
Less than 20 h	38	6.2
20–40 h	335	54.5
41–60 h	181	29.4
61–80 h	32	5.2
More than 80 h	1	0.2
Substance Use		
Drinking	442	71.9
Marijuana use	29	4.7
Other drug	80	13

Note: Demographic variables are provided for the sample that also responded to substance use items (*n* = 615). Age and work schedule were reported as ordinal variables. Participants responded to each substance use item separately and by design could report use of multiple substances.

**Table 2:** Descriptive statistics and bivariate correlations among study variables.

	Variable	Median (Q1, Q3)	1	2	3	4	5	6	7	8
1	Age ^†^									
2	Stress	6.00 (5.00, 7.50)	−0.16							
3	Sleep	5.00 (4.00, 7.00)	0.10	−0.43						
4	Secondary Trauma	2.00 (1.00, 3.00)	−0.15	0.28	−0.23					
5	Exhaustion	11.00 (8.00, 13.00)	−0.15	0.51	−0.39	0.36				
6	Depression	6.00 (2.00, 9.00)	−0.13	0.53	−0.53	0.35	0.57			
7	PTSD	10.00 (8.00, 14.00)	−0.14	0.42	−0.36	0.42	0.51	0.71		
8	Anxiety	5.00 (2.00, 9.00)	−0.21	0.61	−0.42	0.39	0.56	0.75	0.73	
9	Workplace	28.00 (22.00, 34.00)	−0.07 (ns)	0.30	−0.25	0.36	0.37	0.49	0.54	0.51
	Cognitive Failure									

Note: Descriptive statistics of variables included for hypothesis testing (*n* = 613). Ordinal scales are reported with median and first and third quartile (Q1, Q3). Spearman rho bivariate correlations are reported; all correlations were significant at *p* < 0.05 except where indicated (ns). ^†^ Age was recoded into two groups for analysis, for which the median is not informative.

**Table 3:** Summary of indirect effects of reported increased substance use on workplace cognitive failure.

	Indirect Effect			
Mediator	Unstandardized Coeff.	Sobel z-Test (*p*-Value)	95% CI (LL, UL)	% of Cumulative Effect
PTSD	**0.306**	**2.84 (0.005)**	**0.095, 0.516**	**31.10**
Anxiety	**0.191**	**2.01 (0.045)**	**0.004, 0.379**	**19.41**
Depression	0.139	1.79 (0.073)	−0.013, 0.292	14.13
Secondary Trauma	**0.123**	**2.28 (0.023)**	**0.017, 0.229**	**12.50**
Exhaustion	0.053	0.98 (0.327)	−0.053, 0.159	5.39
Sleep	−0.022	−0.56 (0.574)	−0.100, 0.056	2.24
Stress	0.017	0.56 (0.574)	−0.043, 0.078	1.73

Note: Indirect effects as tests of mediation are reported from the path model that included age as a covariate and inter-correlations among mediators. Each mediated path is reported with an unstandardized coefficient, statistical significance (bolded for emphasis) and 95% confidence intervals lower limit and upper limit (95% CI; LL, L). The proportion of the cumulative effect is calculated from the absolute total effect of reported increased substance use on workplace cognitive failure including the direct effect.

## Data Availability

The data presented in this study are available on request from the corresponding author.
